# Cell-free methylation markers with diagnostic and prognostic potential in hepatocellular carcinoma

**DOI:** 10.18632/oncotarget.14115

**Published:** 2016-12-23

**Authors:** Chang-Yi Lu, Shih-Ya Chen, Hui-Ling Peng, Pu-Yeh Kan, Wan-Chi Chang, Chia-Jui Yen

**Affiliations:** ^1^ Biomedical Technology and Device Research Labs, Industrial Technology Research Institute, Hsinchu, Taiwan; ^2^ Division of Hematology and Oncology, Department of Internal Medicine, National Cheng Kung University Hospital, College of Medicine, National Cheng Kung University, Tainan, Taiwan

**Keywords:** hepatocellular carcinoma, DNA methylation, marker, microRNA, diagnosis

## Abstract

Hepatocellular carcinoma (HCC) is a highly malignant tumor with poor prognosis and high mortality. There is a dearth of effective early diagnostic tools, so liver resection surgery and liver transplantation are the only effective medical treatments. The most commonly used marker for HCC detection is serum alpha fetoprotein (AFP), which has low sensitivity and specificity. Because aberrant DNA methylation of genes and miRNAs occurs early in most cancers, we explored whether circulating methylation markers could be promising clinical tools for HCC diagnosis. Using a whole-genome approach, we identified many hyper-methylated miRNAs in HCC. Furthermore, three abnormally methylated genes and one miRNA were combined to establish a methylation predictive model and tested for its diagnostic and prognostic potential in HCC. Using plasma samples, the predictive model exhibited high sensitivity and specificity (> 80%) for HBV-related HCC. Most importantly, nearly 75% of patients who could not be diagnosed with AFP at 20 ng/mL were detected by this model. Further, the predictive model exhibited an exceedingly high ability to predict 5-year overall survival in HCC patients. These data demonstrate the high diagnostic and prognostic potential of methylation markers in the plasma of HCC patients.

## INTRODUCTION

Hepatocellular carcinoma (HCC) is the fifth most common cancer worldwide and the third leading cause of cancer deaths, with nearly 746,000 deaths and 782,000 new cases reported annually. HCC is a rapidly progressing, highly malignant tumor with poor prognosis and high mortality. The incidence of HCC varies widely with the geographic location due to variations in exposure to hepatitis B virus (HBV) and hepatitis C virus (HCV). Apart from HBV and HCV infections, liver cirrhosis present as a major risk for HCC.

Also, HCC is relatively chemotherapy resistant with none of the current chemotherapeutic agents capable of improving overall survival. Therefore, surgical intervention that includes partial liver resection and liver transplantation remains the only realistic treatment for HCC. However, only fewer than 30% of HCC patients are eligible for surgery due to advanced stage diagnosis and occurrence of multiple lesions on the cirrhotic or fibrotic liver. Therefore, to improve overall survival of HCC patients, it is imperative that the diagnostic methods be improved to detect at an early stage so that effective treatment can be rendered to the patients.

The serum alpha-fetoprotein (AFP) remains the most widely used marker for HCC screening and surveillance inspite of its poor sensitivity and specificity. Elevated AFP levels can also be due to non-HCC factors like chronic liver ailments such as cirrhosis and hepatic inflammation and other cancer types like non-seminomatous germ cell tumors and gastrointestinal cancers [1]. However, although AFP is poor for early HCC detection, serum AFP levels are efficient in predicting the disease outcome and monitoring tumor progression in AFP-producing HCC patients.

The advent of new cutting-edge genomic and proteomic technologies have opened up newer avenues to explore novel diagnostic and prognostic biomarkers for HCC including various biomolecules such as, proteins, DNA, mRNA, microRNA (miRNA), metabolites, lipids, and abnormally methylated DNA. DNA methylation signatures are not only involved in gene regulation during embryonic development, X-chromosome inactivation, imprinting, and the suppression of parasitic DNA sequences [2], but also in cancer cells. DNA methylation is catalyzed by a family of DNA methyltransferases that add a methyl group to the carbon-5 position of cytosine residues in CpG dinucleotides. It is observed that DNA methylation of the promoter or the 5′ region of the CpG islands results in transcriptional repression of downstream genes [3]. Increasing evidence has shown that DNA methylation is not only a crucial mechanism for downregulating tumor suppressor genes but also for tumor suppressor miRNAs in many cancer cells [4]. The miRNAs represent small noncoding RNAs of approximately 22 nucleotides that bind to the 3′UTR of target gene transcripts and regulate gene expression by cleaving mRNAs or inhibit protein translation. To date, more than 1000 miRNAs have been discovered and predicted to regulate nearly 60% of mammalian genes [5]. A single miRNA is capable of regulating the expression of hundreds of genes and therefore function as an upstream regulator of many crucial pathways. Recent evidence suggests that aberrant methylation of miRNAs occurs at the very early stage in tumor progression [6]. Therefore, aberrantly expressed miRNAs regulated by DNA methylation could be useful for early cancer diagnosis. In this study, our aim was to analyze the diagnostic and prognostic potential of circulating hyper-methylated miRNAs and tumor suppressor genes in HCC using a whole genome approach. Further, we aimed to test the clinical sensitivity and specificity of the methylation markers using patient plasma samples and compare their efficacy with the existing AFP model for diagnosis and prognosis.

## RESULTS

### Identification of novel miRNAs regulated by DNA methylation in HCC cells

**Figure 1:**
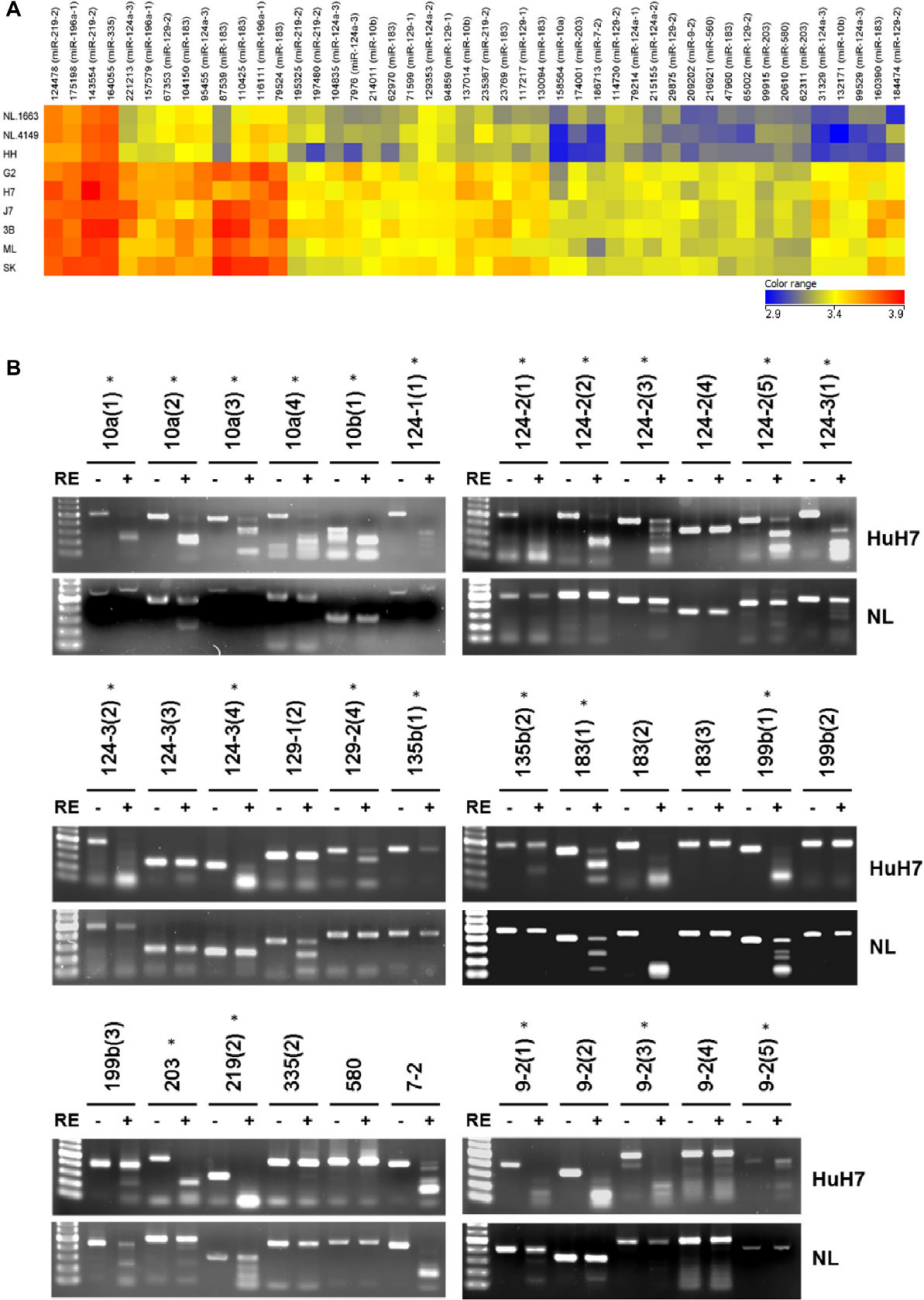
Differential DNA methylation profile of miRNAs in normal livers and HCC cells (**A**) Unsupervised hierarchical cluster analysis of the CpG microarray data from two normal liver tissues (NL-1663 and NL-4149), one normal liver cell (HH) and six HCC cells (G2, H7, J7, 3B, ML, SK). Each row represents a sample, and each column represents a miRNA. The color score (bottom right) depicts the log-transformed and normalized intensity. Hyper-methylated and hypo-methylated miRNAs are indicated in red and blue, respectively. The abbreviations of HCC cell lines are as follows: G2, HepG2; H7: HuH7; 3B, Hep3B; ML, Mahlavu; SK, SK-Hep-1. (**B**) The results of COBRA in HuH7 and normal liver (NL). Following PCR amplification of sodium bisulfite-converted DNA, PCR products were incubated with or without restriction enzyme (RE) as indicated by plus or minus sign. Different regions of the same miRNA are shown as parentheses. Lane 1, 100-bp DNA marker. Stars represent significant hypermethylation in HuH7.

In this study, a genome-wide approach was used to identify novel miRNAs regulated by DNA methylation. The six HCC cell lines (HepG2, HuH7, J7, Hep3B, Mahlavu and SK-Hep-1), two normal liver tissues (NL-1663 and NL-4149) and one normal liver cell line (HH) were subjected to differential methylation hybridization (DMH) by using CpG microarray. The log-transformed intensity values were normalized and analyzed by unsupervised hierarchical clustering. The selection criteria for candidate miRNAs was that (1) all the six HCC cell lines should demonstrate higher intensities than the non-HCC control cell lines (NL-1663, NL-4149 and HH) and (2) statistical significance was demonstrated by a *P value* of less than 0.05 based on the two-sample *t*-test. We found 18 miRNAs (47 probes) hyper-methylated in the six HCC cell lines compared to normal liver cells and tissues (Figure 1A). The hyper-methylated miRNAs included miR-10a, miR-10b, miR-124-1, miR-124-2, miR-124-3, miR-129-1, miR-129-2, miR-135b, miR-183, miR-196a-1, miR-199b, miR-203 miR-219-2, miR-335, miR-560, miR-580, miR-7-2 and miR-9-2. Nine of the eighteen miRNAs (miR-10a, miR-10b, the miR-124 family, miR-129-2, miR-203, miR-335 and miR-9-2) had previously been reported to be hypermethylated in HCC, whereas two miRNAs, miR-199b and miR-219-2 had been associated with epigenetic suppression in other types of cancer [7, 8]. The epigenetic regulation of the remaining seven miRNAs (miR-129-1, miR-135b, miR-183, miR-196a-1, miR-560, miR-580 and miR-7-2) was reported for the first time in this study.

### Validation of hypermethylated miRNAs from CpG microarray through COBRA

To validate the results obtained from differential methylation hybridization (DMH), we selected 16 miRNA candidates (previously reported with HCC: miR-10a, miR-10b, miR-124-1, miR-124-2, miR-124-3, miR-129-2, miR-203, miR-335 and miR-9-2; not reported with HCC: miR-129-1, miR-135b, miR-183, miR-199b, miR-219, miR-580 and miR-7-2) for combined bisulfate restriction analysis (COBRA) (Figure 1B). Multiple, probable methylation sites (indicated in parentheses) were probed in several miRNAs to determine their methylation status. For example, four regions were probed by COBRAin the CpG islands of miR-10a and designated as miR-10a (1), miR-10a (2), miR-10a (3) and miR-10a (4), respectively. In total, 35 CpG islands in 16 miRNAs were analyzed by COBRA in hepatoma cells (HuH7) and normal liver tissues (NL). The PCR products of 20 CpG island regions in multiple miRNAs that were amplified from sodium bisulfite-treated DNA (miR-10a (1), miR-10a (3), miR-10a (4), miR-10b (1), miR-124-1 (1), miR-124-2 (1), miR-124-2 (2), miR-124-2 (3), miR-124-2 (5), miR-124-3 (1), miR-124-3 (2), miR-124-3 (4), miR-129-2 (4), miR-135b (1), miR-135b (2), miR-203, miR-9-2 (1), miR-9-2 (2) , miR-9-2 (3), and miR-9-2 (5)), were digested with methylation-sensitive restriction enzymes in HuH7, but remained undigested in normal liver tissue samples. In addition, the PCR products of miR-10a (2), miR-199b (1), and miR-219 (2) showed complete digestion in Huh7 and partial digestion in normal liver tissues. In conclusion, 23 CpG islands in 10 miRNAs were found to be hyper-methylated in tumor cells compared to adult normal liver tissues.

### Methylation status of miRNA and gene candidates in HCC clinical tissues

**Figure 2:**
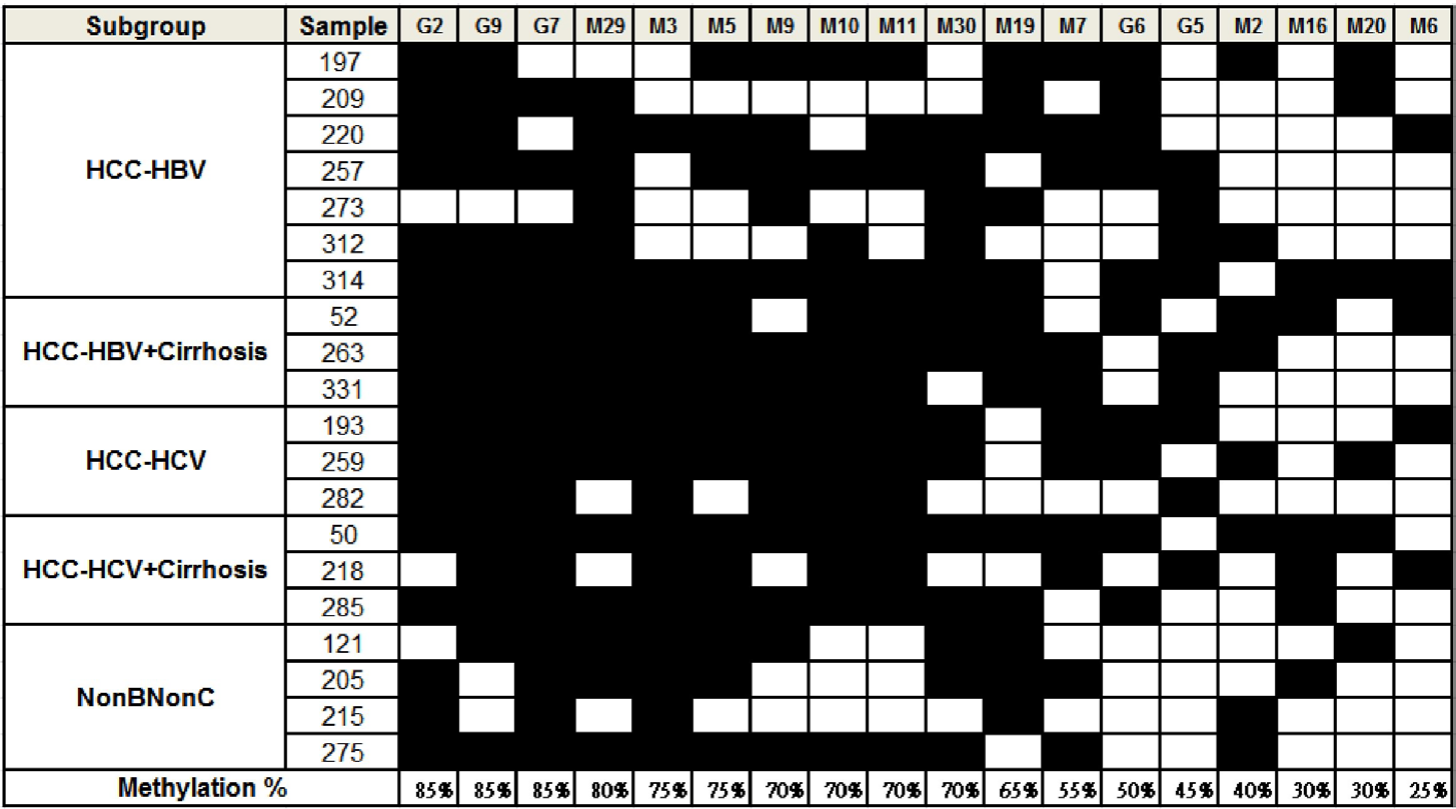
Methylation status of candidate miRNAs and genes in HCC tissues HCC samples were classified into 5 groups as shown in the first column. The filled squares represent the higher methylation level in tumor and the open squares represent the higher methylation level in adjacent normal tissue. The percentage of methylated samples (tumor vs. adjacent normal) was calculated and shown at the bottom. The codes are as follows: G2: RASSF1A, G9: RUNX3, G7: APC, M29: miR-589, M3: miR-10b (C), M5: miR-203, M9: miR-124-3 (A), M10: miR-124-3 (B), M11: miR-124-3 (D), M30: miR-589 (C), M19: miR-647, M7: miR-129-2, G6: COX2, G5: CDKN2A, M2: miR-10b (A), M16: miR-671, M20: miR-335, M6: miR-339B.

Having identified epigenetically regulated miRNAs in HCC, we searched literature to identify potential candidate genes that are silenced by DNA methylation that could serve as useful biomarkers for HCC diagnosis. The methylation status of 18 candidates, including five genes (RASSF1A, RUNX3, APC, COX2 and CDKN2A) and nine miRNAs (miR-10b, miR-124-3, miR-129-2, miR-203, miR-335, miR-339B, miR-589, miR-647 and miR-671), was examined in 20 HCC tissue pairs (Figure 2). We classified the samples into five groups namely, HBV-related HCC, HBV-related HCC with cirrhosis, HCV-related HCC, HCV-related HCC with cirrhosis and HCC without HBV or HCV infection. Our data showed that 13 out of 18 candidates that included four genes (RASSF1A, RUNX3, APC, and COX2) and six miRNAs (miR-589, miR-10b, miR-203, miR-124-3, miR-647 and miR-129-2), were hyper-methylated in more than50% of tumor samples compared to the adjacent normal tissues. However, hyper-methylation of the tumor suppressor gene CDKN2A was observed in only 9 of the 20 HCC tissues (45%) in this study. In addition, we observed that two regions in miR-10b, designated ‘A’ and ‘C’ showed variable methylation percentages (75% for A and 40% for C) suggesting independent regulation of methylation at different CpG islands. Overall, both the COBRA and the CpG microarray data were consistent for the methylation status of candidate genes and miRNAs in the HCC cells and tissues.

### Methylation levels of potential candidates in HCC plasma samples

Since the criteria for a diagnostic and prognostic HCC marker was the ability to be quantified in body fluids, we evaluated the hyper-methylation status of 13 candidates previously found in more than 50% of HCC tissues by quantitative methylation-specific PCR using plasma samples. Towards this, plasma samples from eight patient groups that were diagnosed either with chronic hepatitis B, chronic hepatitis C, chronic hepatitis B with cirrhosis, chronic hepatitis C with cirrhosis, HBV-related HCC, HCV-related HCC, HBV-related HCC with cirrhosis or HCV-related HCC with cirrhosis were compared with those from healthy donors.

**Figure 3:**
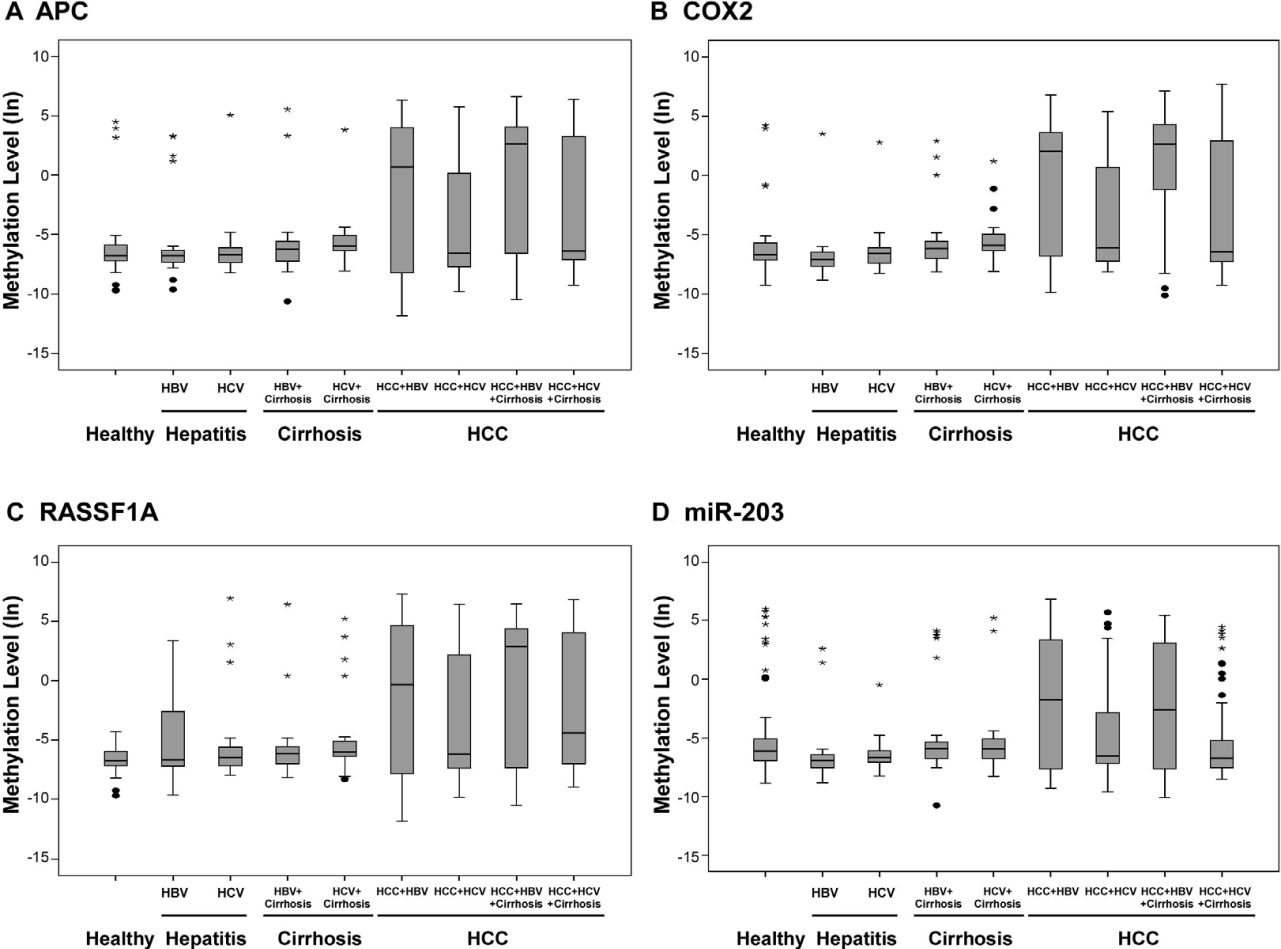
Methylation levels of candidate genes and miRNAs in clinical plasma samples Methylation levels of (**A**) APC, (**B**) COX2, (**C**) RASSF1A, and (**D**) miR-203 were determined by qMSP in plasma samples from healthy controls (*n* = 50) and patients with hepatitis (including hepatitis B and hepatitis C; *n* = 47), hepatitis with cirrhosis (including hepatitis B and hepatitis C; *n* = 57) and HCC (HBV-related, HBV-related with cirrhosis, HCV-related and HCV-related with cirrhosis; *n* = 203). Methylation levels were transformed by log 2 and depicted by box plots. Boxes extend from 25th to 75th percentiles and are divided by a solid line that represents the median of each group and a diamond that represents the mean of each group. Whiskers extend from the 5th to the 95th percentiles. Each outlier is denoted by a dot. F test was used to determine statistical significance.

We observed that four candidates, namely, APC, COX2, RASSF1A, and miR-203 showed significant hyper-methylation levels in HCC than in non-cancerous subgroups (*P* < 0.0001; Figure 3). The hyper-methylation level of the four candidates was greatly elevated in the HBV-related HCC subgroups (with and without cirrhosis) than in the HCV-related HCC subgroups (with or without cirrhosis). This suggested that the methylation status of APC, COX2, RASSF1A and miR-203 have great diagnostic potential in HBV-related HCC.

### Methylation predictive model using four methylation markers for diagnosis of HBV-related HCC

**Figure 4:**
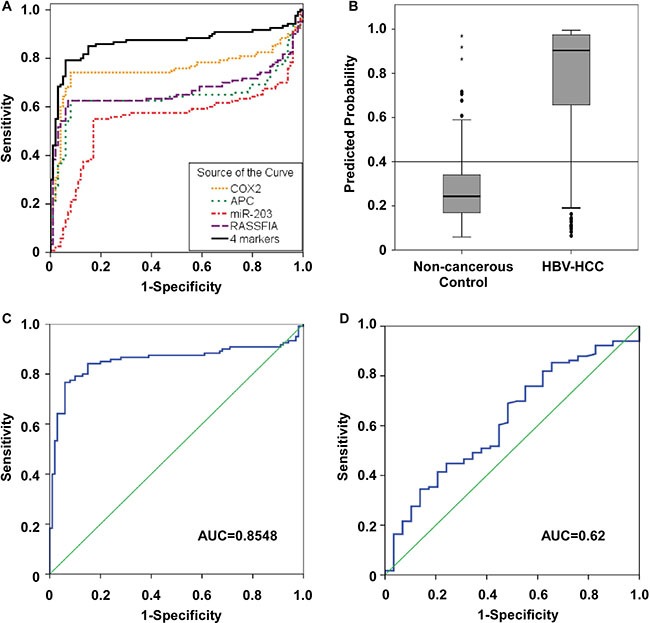
Receiver operator characteristic (ROC) curves for the diagnosis of HBV-related HCC versus non-cancerous control (**A**) ROC curve analysis for APC [AUC=0.644], COX2 (AUC = 0.758), RASSF1A (AUC = 0.666), miR-203 (AUC = 0.55) and the four candidate combination (AUC = 0.865). (**B**) A MPM-B cut-off value of 0.4 for differentiating between HBV-related HCC and non-cancerous control. (**C**) ROC curve for leave-one-out cross-validation of MPM-B (AUC = 0.8548). (**D**) ROC curve for serum AFP to discriminate between HBV-related HCC and non-cancerous control (AUC = 0.62).

We conducted ROC curve analyses to further explore if the methylation status of the four candidates could distinguish HBV-related HCC from healthy donors, patients with chronic hepatitis B and patients with chronic hepatitis B and cirrhosis. The AUC (area under the curve) values for APC, COX2, RASSF1A, and miR-203 were 0.644, 0.758, 0.666 and 0.55, respectively (Figure 4A). We combined the four candidates to form a methyl predictive model B (MPM-B) and tested the diagnostic potential using a stepwise logistic regression algorithm. Our data showed that the MPM-B achieved a sensitivity of 84.2%, a specificity of 83% and an AUC of 0.87 with false positive rate (FPR) of 14.4% and false negative rate (FNR) of 18.6% for HBV-related HCC (Table 1). Also, we found that HBV-related HCC can be clearly distinguished from the controls at a cut-off value of 0.4 (*P* < 0.05; Figure 4B). Furthermore, the leave-one-out cross-validation (LOOCV) showed AUC of 0.855, sensitivity of 83.3%, and specificity of 83.0% for the same plasma samples (Figure 4C). Therefore, our analysis clearly showed the stability and the reliability of the MPM-B for diagnosis of HBV-related HCC.

**Table 1:** Area under curve (AUC), sensitivity, specificity, false positive rate (FPR), and false negative rate (FNR) of MPM-B and AFP for the diagnosis of HBV-related HCC

	MPM-B	AFP = 20	AFP = 12.15
AUC	0.87	0.56	0.62
Sensitivity, %	84.2	50.9	55.2
Specificity, %	83.0	62.1	51.7
FPR, %	14.4	15.7	17.9
FNR, %	18.6	76	77.6

**Figure 5:**
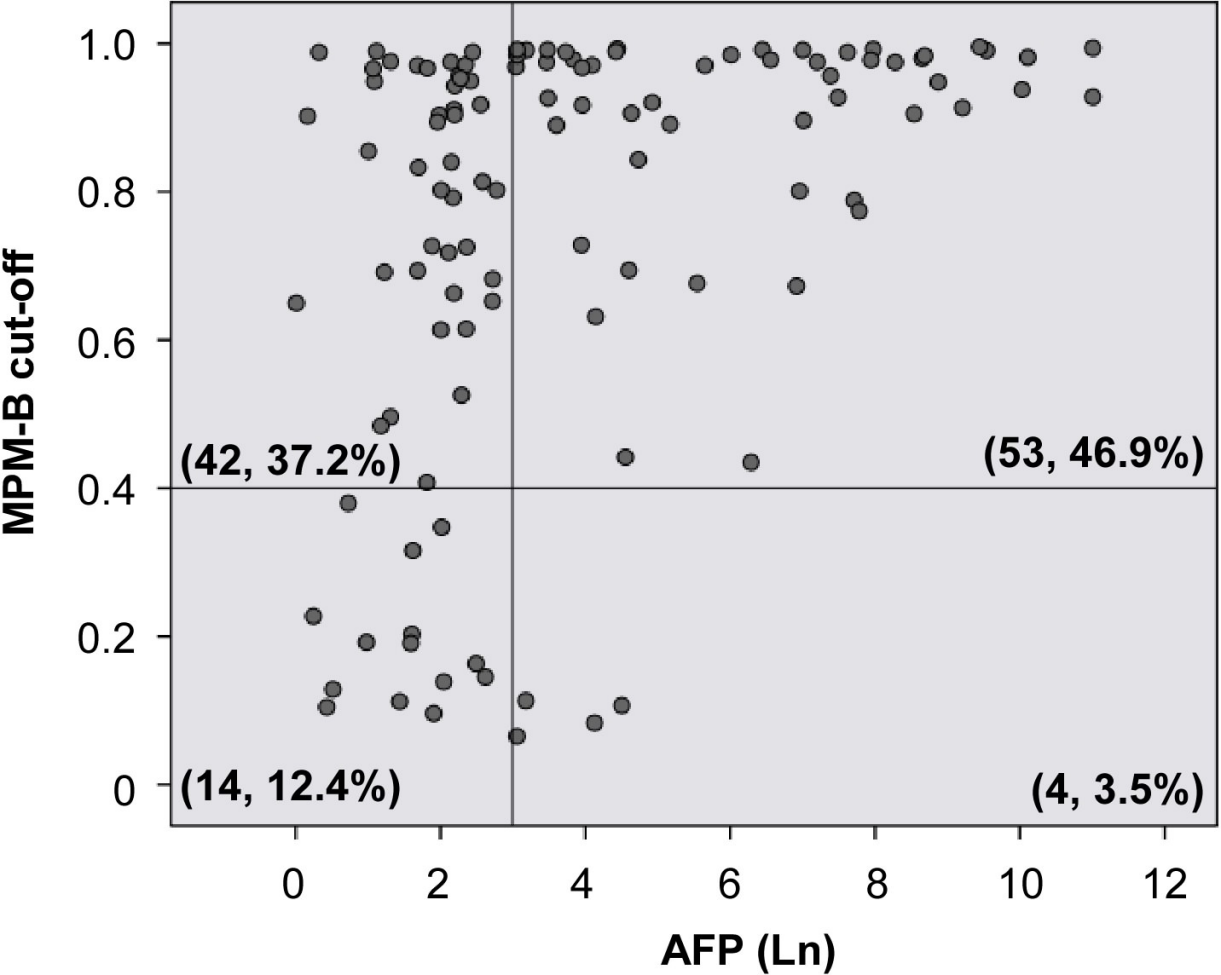
Comparison of sensitivity scores of AFP level and MPM-B in HBV-related HCC patients Scatter plot shows the distribution of 113 HBV-related HCC patients with the cut-off values of AFP (y-axis) and MPM-B (x-axis) as indicated. Serum AFP level was shown as natural log transformed. Each circle represents an individual HBV-related HCC case. The vertical reference line depicts the 20 ng/ml AF *P value*. A horizontal reference line depicts the MPM-B score of 0.4. The number of cases and the percent of the total HBV-related HCC in each of four areas are as indicated.

Further, we analyzed AFP, which is the most commonly used serum biomarker in the HBV-related HCC patients who were used for MPM-B assessment. At a cutoff value of 20 ng/mL, the sensitivity and specificity of AFP were 50.9% and 62.1%, respectively, with an FPR of 15.7% and an FNR of 76% (Table 1). When we performed the ROC analysis at 12.15ng/mL, AFP had a sensitivity of 55.2%, a specificity of 51.7%, and an AUC of 0.62 with an FPR of 17.9% and an FNR of 77.6% (Figure 4D and Table 1). We then compared the diagnostic ability of the MPM-B with that of AFP in 113 HBV-related HCC patients and found that only 57 patients (50.44%) had AFP levels higher than 20 ng/mL, whereas, 95 patients (84.1%) were positive for HCC using the MPM-B model (Figure 5). These results demonstrated that the combined methylation marker model had greater diagnostic ability than the currently used AFP detection model for HBV-related HCC.

### Assessing the rognostic ability of the methylation predictive model

**Figure 6:**
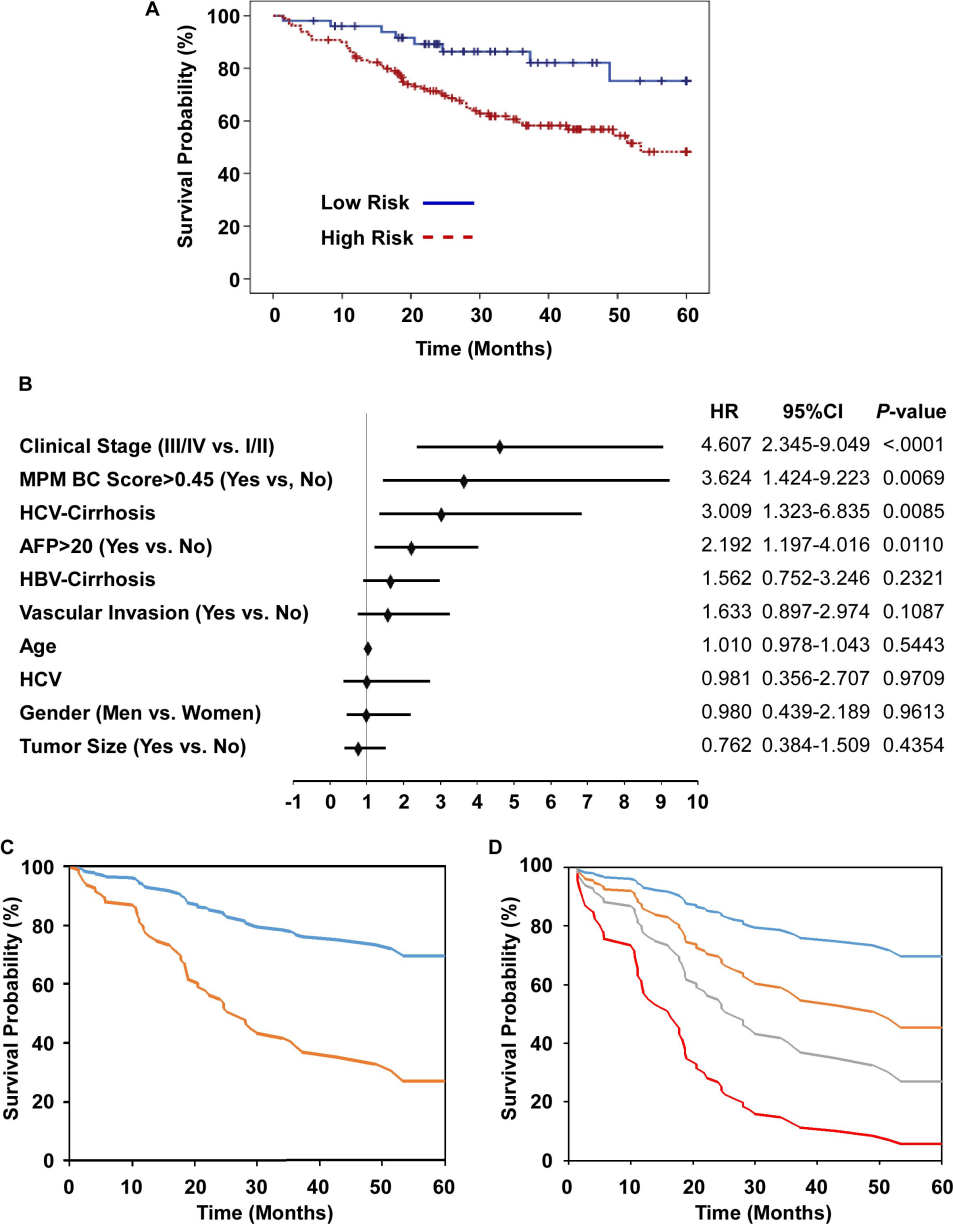
Prognostic potential of risk factors, AFP and MPM-BC in HCC patients (**A**) Kaplan-Meier analysis of overall survival for HCC patients classified as high- and low- risk groups according to MPM-BC with cutoff value of 0.45. (**B**) Multivariate Cox proportional-hazards model for overall survival. HR, hazard ratio; *, HBV-related HCC as a reference. (**C**) Covariate-adjusted survival curve stratified by high (orange) and low (blue) risk groups. (**D**) Covariate-adjusted survival curve stratified by both the AFP levels and the MPM-BC score. Blue line: MPM-BC ≤ 0.45 and AFP ≤ 20 ng/mL; Orange line: MPM-BC ≤ 0.45 and AFP > 20 ng/mL; Gray line: MPM-BC > 0.45 and AFP ≤ 20 ng/mL; Red line: MPM-BC > 0.45 and AFP > 20 ng/mL.

Having tested the diagnostic efficacy of the methylation markers for HCC, we analyzed the prognostic capability for the methylation prediction model in a retrospective cohort of 241 HCC patients with chronic HBV with or without HCV infection (180 patients survived to the end of the follow-up period). The logistic regression model was used to establish a methylation predictive model BC (MPM-BC) and the relationship between clinico-pathological characteristics and overall survival was statistically determined by the Log-Rank test. Based on the univariate analysis, the significant prognostic factors for survival included cirrhosis (*P* = 0.0086), the histologic grade (*P* = 0.0382), AFP (*P* < 0.0001), the pathological stage (*P* = 0.0054), the clinical stage (*P* < 0.0001), vascular invasion (*P* < 0.0001) and the MPM-BC (*P* = 0.0052) (Table 2). Further, the HCC patients were subdivided into high- and low- risk groups based on the MPM-BC cutoff value of 0.45. Based on the Kaplan-Meier survival curves, the 5-year survival rates for the high- and the low-risk groups was 48.3% and 75.2%, respectively (Figure 6A). We further analyzed the various clinico-pathological factors like age, gender, AFP, vascular invasion, tumor size, the clinical stage, the viral cirrhosis group and the MPM-BC by using the multivariate Cox proportional hazard model and found that the clinical stage (*P* < 0.0001), MPM-BC (*P* = 0.0069), the viral cirrhosis group (*P* = 0.0085) and AFP (*P* = 0.011) were statistically significant for overall survival (Table 3). The Cox proportional hazards model also showed that the Clinical stage III/IV (HR, 4.607; 95% CI, 2.345–9.049), the HCV-related HCC with cirrhosis subgroup (HR, 3.009; 95% CI, 1.325–6.835), > 20 ng/mL AFP (HR, 2.192; 95% CI, 1.197–4.016) and the high risk group of MPM-BC (cut-off value > 0.45) (HR, 3.624; 95% CI, 1.424–9.223) were associated with high mortality rates (Figure 6B). Additionally, we analyzed the overall survival of the high- and low-risk groups stratified by the MPM-BC using the fitted Cox proportional hazard model and Breslow estimate of the background hazard. The co-variate adjusted survival curves showed that the average 5-year survival probability of the high- and the low-risk groups were 26.94% and 69.63%, respectively (Figure 6C). Comparatively, patients with MPM-BC ≤ 0.45 and AFP ≤ 20 ng/mL exhibited the survival rate of 69.63%, whereas, patients with MPM-BC > 0.45 and AFP > 20 ng/mL had a low survival rate of 5.64% (Figure 6D). Also, since the patients with MPM-BC ≤ 0.45 and AFP > 20 ng/mL exhibited a relatively higher 5-year survival rate than that of the patients with MPM-BC > 0.45 and AFP ≤ 20 ng/mL as shown in Figure 6D, it showed greater prognostic potential of MPM-BC for HCC patients.

**Table 2:** Univariate analysis of variables potentially predictive of survival in HCC

Variable	Number of Persons at Risk	Death		*P*-value
		Number	Percent	
Gender				0.5771
Female	42	12	28.57	
Male	138	49	35.51	
Groups				0.0664
HBV	77	19	24.68	
HCV	27	6	22.22	
HBV+cirrhosis	38	20	52.63	
HCV+cirrhosis	34	14	41.18	
Histologic grade				0.0382
M	98	27	27.55	
P	13	5	38.46	
W	26	2	7.69	
Tumor Size (cm)				0.3251
≤ 5	133	42	31.58	
> 5	47	19	40.43	
AFP (ng/ml)				< .0001
≤ 20	96	20	20.83	
> 20	84	41	48.81	
Pathological Stage				0.0054
Stage I, II	161	49	30.43	
Stage III, IV	19	12	64.16	
Clinical Stage				< .0001
Stage I, II	143	35	24.48	
Stage III, IV	37	26	70.27	
Vascular invasion				< .0001
No	130	30	23.08	
Yes	41	25	60.98	
MPM-BC				0.0052
≤ 0.45	50	8	16.00	
> 0.45	130	53	40.77	

**Table 3:** Cox proportional hazards analysis of prognostic parameters in HCC

Parameters	Estimate	S.E.	Chi-square	Pr > ChiSq	Hazard Ratio	95% C.I.
Age	0.01006	0.01658	0.3676	0.5443	1.010	(0.978, 1.043)
Gender	−0.01987	0.40992	0.0023	0.9613	0.980	(0.439, 2.189)
(Men vs. Women)						
AFP > 20	0.78500	0.30877	6.4635	0.0110	2.192	(1.197, 4.016)
(Yes vs. No)						
Vascular Invasion	0.49046	0.30580	2.5723	0.1087	1.633	(0.897, 2.974)
(Yes vs. No)						
Tumor size > 5 cm	-0.27213	0.34892	0.6083	0.4354	0.762	(0.384, 1.509)
(Yes vs. No)						
Clinical Stage	1.52756	0.34444	19.6680	< .0001	4.607	(2.345, 9.049)
III/IV vs. I/II						
Viral cirrhosis group HBV-HCC (reference group)	1	—	—	—	—	— —
HCV-HCC	−0.0189	0.51779	0.0013	0.9709	0.981	(0.356, 2.707)
HBV/cirrhosis-HCC	0.446	0.37326	1.4277	0.2321	1.562	(0.752, 3.246)
HCV/cirrhosis-HCC	1.10165	0.4186	6.9259	0.0085	3.009	(1.325, 6.835)
MPM-BC > 0.45 vs. ≦0.45	1.28755	0.47663	7.2974	0.0069	3.624	(1.424, 9.223)

## DISCUSSION

Aberrant DNA methylation of genes and miRNAs is associated with many aspects of tumor biology and is believed to occur at early stages of carcinogenesis. DNA methylation signatures can be detected in body fluids, such as whole blood, plasma, serum, saliva, and urine and hence, are more amenable for clinical purposes. RASSF1A, APC and COX2 are methylated tumor suppressor genes that are associated with HCC. RASSF1A is a key regulator of the cell cycle and its aberrant expression is associated with many types of cancers. Detection of hyper-methylated RASSF1A in a premalignant liver has suggested its involvement in early stages of hepatocarcinogenesis [9]. Hypermethylated RASSF1A was detected by qPCR analysis after digestion of serum samples with a methylation-sensitive restriction enzyme in 93% of HCC patients and 58% of HBV carriers compared to 8% of healthy controls. A two-step methylation sensitive PCR (MSP) analysis showed hyper-methylation of APC in 16 of the 26 (61.5%) HCC plasma samples compared to 2 out of16 (12.5%) in liver cirrhosis plasma samples suggesting a role for hyper-methylation in HCC [10]. COX2 is a prostaglandin synthase that produces prostanoids like thromboxane and prostacyclin in response to pro-inflammatory cytokines, growth factors, carcinogens and other external stimuli [11]. Enhanced COX2 levels and upregulated prostaglandin pathway promote carcinogenesis by altering angiogenesis, cell proliferation, and apoptosis. COX2 promoter hyper-methylation has been reported in colorectal cancer, gastric cancer and HCC although its over-expression has also been reported in most cancers [12]. In HCC, COX2 methylation was found in 25% of 48 tumor tissues compared to 4.2% in corresponding noncancerous tissues [13]. Another study showed that although COX2 methylation was absent in normal livers, cirrhotic livers and low-grade dysplastic nodules, a step-wise increase was observed from high-grade dysplastic nodules to advanced HCC [14]. These findings highlighted the diagnostic potential of methylation markers in HCC. Our data showed higher COX2 methylation in the HBV-related HCC subgroup than in the healthy donors and chronic hepatitis B patients. A highly significant AUC value of 0.758 was determined for COX2. However, since previous studies have indicated that HBV promotes COX2 overexpression through promoter demethylation and transcription factor recruitment [15], the role of COX2 expression regulated by HBV in hepatocarcinogenesis should be further elucidated.

Large scale microRNA expression profiles have highlighted aberrant expression of miRNAs in many cancer types. MicroRNA expression is highly regulated by epigenetic mechanisms like DNA methylation and histone modification. Based on our CpG microarray results, six out of nine miRNAs (miR-589, miR-10b, miR-203, miR-124-3, miR-647 and miR-129-2) were found to be hypermethylated in more than 50% of HCC tissues analyzed. Since previous studies have shown hyper-methylation of miR-203, miR-124-3 and miR-129-2 in HCC, they may function as tumor suppressor miRNAs [16–18].

The role of miR-10b is highly complex and possibly depends on the stage of cancer being analyzed as illustrated in many previous studies in various cancer types. Downregulation of miR-10b expression was reported in primary breast cancer [19]. Similarly, miR-10b was found to be repressed by promoter hypermethylation in human gastric cancer cells suggesting a tumor suppressor function [20]. However, another study showed that miR-10b was highly expressed in metastatic breast cancer cells and actively promoted cell migration and invasion that was contradictory to the previous finding [21]. The functional diversity of miR-10b in metastatic or non-metastatic tumors was also reported in HCC. A genome-wide study showed that miR-10b was hyper-methylated in primary liver tumors [22]. However, miR-10b promoted cell proliferation, migration and invasion in metastasizing HCC by regulating RhoC, urokinase-type plasminogen activator receptor, matrix metallopeptidase 2 and matrix metallopeptidase 9 [23].

Regarding miR-589, it has been postulated to regulate the epithelial-mesenchymal transition in peritoneal mesothelial cells, though not directly associated with carcinogenesis [24]. Further, altered miR-647 expression has been detected in prostate and metastatic gastric cancers [25, 26]. However, since the dysregulation of miR-589 and miR-647 is not reported in HCC, our findings are the first in regard to these two miRNAs, although further studies are essential to identify their biological role in HCC development.

It is challenging to diagnose HCC at an early stage with conventional detection tools. Serum AFP is the most established marker used in clinical screening for HCC despite its poor sensitivity at high cut-off values. Surveillance of HCC patients is commonly performed using the serum AFP in combination with radiographic image, such as computed tomography or ultrasonography. The fetal yolk sac and fetal liver generate high levels of AFP, and malignant tumors derived from the hepatic diverticulum may also elevate the serum AFP levels, including stomach, pancreas, and biliary tract [27]. In addition, chronic hepatitis or cirrhosis raise AFP in 20% and 40% of patients, respectively [28]. Normal AFP levels are present in as many as 30% of patients at time of diagnosis and usually remain low, even with advanced HCC [29]. AFP > 400 ng/ml is considered diagnostic for HCC. The specificity of AFP is close to 100% but the sensitivity which falls below 20%. Using AFP 20 ng/ml as the cut-off point, the sensitivity could rise to 88–90%; however, the specificity declined to 55–60% [30]. In our study, the sensitivity and specificity of AFP in HBV-related HCC diagnosis are 50.9% and 62.1%, respectively. By contrast, in the same patient population, the sensitivity and specificity of MPM-B reach to 84.2% and 83%, respectively. Moreover, 75% of AFP-negative HBV-related HCC can be detected by MPM-B (Figure 5). These results show the potential of MPM-B for diagnosis of HBV-related HCC.

Since good prognostic prediction is essential to ascertain the risk and the effectiveness of treatments such as surgical resection and radiotherapy, AFP remains a competent prognostic marker to predict treatment response, and overall survival in HCC patients, in spite of its poor diagnostic ability. AFP levels have been shown to rapidly and dramatically decrease after curative hepatic resection and increase upon recurrence after surgical treatment as shown in five out of six patients analyzed previously [31]. A recent analysis of 108 HCC patients that were divided into three groups based on AFP levels (AFP-negative group (AFP ≤ 20 ng/mL), a lower AFP group (AFP = 20–400 ng/mL), and a higher AFP group (AFP > 400 ng/mL)) showed that the AFP-negative group had a lower post-operative two-year recurrence rate and higher 18- and 24-month survival rates than the other two groups (*P* < 0.05) [32]. Similarly, a retrospective study of 2253 patients that underwent orthotopic liver transplants indicated that the serum AFP levels were an independent prognostic predictor of orthotropic liver transplant outcomes [33]. Therefore, the serum AFP level can provide useful information towards prioritizing patients on the waiting list for liver transplantation. Also, serum AFP has been useful in determining and monitoring the response of HCC patients undergoing systemic chemotherapy and loco-regional therapies, including trans-arterial chemo-embolization (TACE) and radio-embolization (yttrium-90 [Y90]) [34, 35]. Our data is consistent with previous data that AFP levels are a good predictor of overall survival. However, our data shows that the MPM-BC model has greater sensitivity and accuracy than theft in predicting the 5 year overall survival rates.

In conclusion, we identified differentially methylated miRNAs, with diagnostic and prognostic potential. We also showed that combining multiple methylation markers (MPM-B) could accurately identify HBV-related HCC from patient plasma samples. Also, we demonstrated the utility of the MPM-BC model as both a diagnostic and a prognostic tool for HCC. In future, a large multicenter cohort study to confirm the predictive value of MPM-BC for HCC and a follow-up study in post-operative patients to analyze if methylation levels decline are necessary to further investigate the potential clinical use of the MPM as a diagnostic and prognostic marker.

## MATERIALS AND METHODS

### Cell culture and patient samples

The liver cell line, HH (ScienCell Research Laboratories) and the hepatoma cell lines, HepG2, HuH-7, J7, Hep3B, Mahlavu and SK-Hep-1, were grown in DMEM at 37°C in a humidified, 5% CO_2_ incubator. Twenty pairs of HCC tissue samples and 357 plasma samples from healthy donors (*n* = 50) and patients that were diagnosed with chronic hepatitis B (*n* = 21), chronic hepatitis C (*n* = 26), chronic hepatitis B with cirrhosis (*n* = 32), hepatitis C with cirrhosis (*n* = 25), HBV-related HCC (*n* = 81), HCV-related HCC (*n* = 30), HBV-related HCC with cirrhosis (*n* = 42) and HCV-related HCC with cirrhosis (*n* = 50), were obtained from National Cheng Kung University (NCKU) Hospital, Tainan, Taiwan. All experimental protocols and study methods were approved by the Institutional Review Board of Human Research of NCKU Hospital. Genomic DNA of adult normal livers (NL-1663 and NL-4149) was purchased from US Biologicals. Human methylated and unmethylated DNA sets that were used as positive and negative controls for quantitative methylation specific PCR were purchased from Zymo Research.

### Differential methylation hybridization (DMH) using CpG island microarrays

DMH was performed as previously described [36]. Briefly, 2 μg of DNA was digested with *Mse*I and ligated to the annealed linkers H-12 (5′-TAATCCCTCGGA-3′) and H-24 (5′-AGGCAACTGTGCTATCCGAGGGAT-3′). The sample was then digested with methylation-sensitive endonucleases *Bst*UI and *Hpa*II, followed by PCR amplification for 20 cycles using the H-24 linker. Amplicons were labeled with the fluorescent dyes Cy5 and applied to Human CpG Island Microarray (Agilent Technologies). Hybridization was carried out at 60°C in a HybChamber (Digilab Genomic Solutions) overnight. Further, the slides were scanned with the GenePix 4000B microarray scanner (Axon) and the intensities of spot images were acquired by the GenePix Pro6.0 (Axon). Microarray data were further analyzed using GeneSpring 13 (Agilent Technologies).

### Combined bisulfite restriction analysis (COBRA)

Genomic DNA (1 μg) from HCC clinical samples, hepatoma cells, and adult normal livers were bisulfite-converted by using the EZ DNA methylation kit (Zymo Research). Bisulfite converted genomic DNA was subjected to polymerase chain reaction (PCR) using the Kapa Sybr Fast qPCR kit (Kapa Biosystems). PCR was performed using 1 μl of converted DNA in a 20 μl PCR reaction containing 0.5 μM of each primer and 1x Kapa Sybr Fast qPCR Master Mix. The PCR condition was as follows: 95°C for 3 min, 40 cycles of 95°C for 3 sec, annealing temperature for 20 sec, 72°C for 10 sec, and final extension at 72°C for 20 sec. Primer and probe sequences are available upon request. Amplified DNA was digested with appropriate restriction enzymes that recognized atleast one CpG site in their recognition sequences. Digested DNA fragments were visualized on 1.5% (w/v) ethidium bromide-stained agarose gels.

### Real-time quantitative methylation analysis

Bisulfite converted DNA from COBRA (described above) was amplified by real-time quantitative methylation-specific PCR (qMSP) using fluorescent probes. Each reaction contained 1x Kapa Probe Fast qPCR Master Mix, 0.5 μM of each primer and 0.25 μM of probe in a total volume of 20 μl. Amplification was performed on the StepOnePlus Real-Time PCR System (Thermo Fisher Scientific). Primer and probe sequences are available upon request. As previously described [37], methylation level was calculated as the difference in Ct value between β-actin and the individual candidates using the following formula: 2^[Ct (β-actin) - Ct (candidate)]^ × 100 for tissue samples or 2^[Ct (β-actin) - Ct (candidate)]^ × 1000 for plasma samples.

### Statistical analysis

Methylation levels of four candidates were subjected to log transformation. One-way ANOVA was used to test the significance of the methylation levels in the nine subgroups, including healthy donors, chronic hepatitis B, chronic hepatitis C, chronic hepatitis B with cirrhosis, chronic hepatitis C with cirrhosis, HBV-related HCC, HCV-related HCC, HBV-related HCC with cirrhosis, and HCV-related HCC with cirrhosis. The logistic regression models were used to establish methyl predictive model B (MPM-B) for diagnosis of HBV-related HCC and the methyl predictive model BC (MPM-BC) for prognosis of HBV- and HCV-related HCC. To assess the diagnostic effects, receiver operating characteristic (ROC) curve analysis was used to estimate the parameters like area under the curve (AUC), cutoff value, sensitivity and specificity. The performance of the model was also evaluated by using leave-one-out cross validation (LOOCV). Univariate COX regression analysis was used to assess the association between each variable and survival. Survival curves were calculated by the Kaplan–Meier method and distributions were compared using the log-rank test. Disease-specific overall survival was calculated from the date of diagnosis until disease-related death or end of follow-up. Cox proportional hazards model was used in multivariate analyses and used to estimate Hazard Ratios (HRs) and their 95% confidence intervals (CIs). Breslow estimates of the survivorship curves at the median of the covariates for different subgroups were computed and plotted. A *P* < 0.05 value was considered statistically significant.
